# Improved Case Detection of Fleaborne Typhus by PCR Testing, Los Angeles County, California, USA, 2022–2024

**DOI:** 10.3201/eid3209.260554

**Published:** 2026-09

**Authors:** Armine Sanosyan, Zuelma A. Contreras, Van Ngo, Michael K. Brown, Nicole M. Green, Lee A. Borenstein, Jill K. Hacker, Will S. Probert, Alexa C. Quintana, Monica P. Haw, Umme-Aiman Halai

**Affiliations:** Los Angeles County Department of Public Health, Los Angeles, California, USA (A. Sanosyan, Z.A. Contreras, V. Ngo, M.K. Brown, N.M. Green, L.A. Borenstein, U.-A. Halai); California Department of Public Health Center for Laboratory Sciences Viral and Rickettsial Disease Laboratory, Richmond, California, USA (J.K. Hacker, W.S. Probert, A.C. Quintana, M.P. Haw).

**Keywords:** *Rickettsia*, *Rickettsia typhi*, Typhus, bacteria, endemic flea-borne, *Rickettsia* infections, vector-borne infections, vector-borne, flea-borne, real-time PCR, epidemiology, public health, Los Angeles County, California, United States

## Abstract

Fleaborne typhus (FBT) is reemerging as a significant cause of febrile illness in southern California, USA. We performed enhanced testing on low-titer *Rickettsia typhi* antibody–positive specimens from patients with FBT-compatible illness reported in Los Angeles County, California, during 2022–2024. We tested 114 specimens by repeat serology and 113 by real-time PCR; 81 (71%) were PCR-positive, and 95 (83%) had higher antibody titers by repeat serology. Most PCR-positive specimens were collected 8–10 days after illness onset, but some were collected up to 15 days after illness onset. All PCR-positive specimens were *R. typhi*–specific. After additional testing, 113 (99%) suspected cases were reclassified as probable or confirmed FBT cases. Our findings indicate that many low-titer seropositive results represent true infections and that use of minimum serology thresholds for public health investigation underestimates surveillance case counts. Expanding commercial molecular testing capacity will improve clinical FBT diagnosis and enhance surveillance accuracy.

Fleaborne typhus (FBT), also referred to as murine or endemic typhus, is a febrile illness spread by fleas infected with the bacterium *Rickettsia typhi*. Infected fleas are often found on mammalian hosts, including rodents, opossums, dogs, and free-roaming cats (*1*,*2*). FBT became a major public health threat in the United States from the 1930s through the early 1940s; thousands of cases were reported annually. Improvements in sanitation, alongside the use of novel rodenticides and synthetic insecticides like DDT, led to a sharp decline in cases by the 1950s (*3*,*4*). Today, most cases are reported in Hawaii, Texas, and southern California; in southern California, the disease is reemerging as a major cause of febrile illness (*5*–*7*). In Los Angeles County (LAC), FBT has increased since 2010; an average of 169 cases were documented per year during 2021–2025, and an all-time high of 220 cases were reported in 2025.

FBT ranges from a mild, self-limited infection to severe or fatal illness. Cases typically manifest with nonspecific symptoms such as fever, myalgia, headache, nausea, vomiting, and, less frequently, a rash (*8*). Severe clinical manifestations are rare but might include complications such as meningoencephalitis, myocarditis, and hemophagocytic lymphohistiocytosis (*9*). Doxycycline is the treatment of choice for FBT and is highly effective (*5*,*10*). Although the overall case-fatality rate for FBT is low (<1% with appropriate doxycycline treatment), fatal outcomes can still occur, especially in patients with delayed care or underlying health conditions (*11*,*12*). In 2022, LAC recorded 3 FBT-associated deaths involving hemophagocytic lymphohistiocytosis, myocarditis, and septic shock in patients with underlying conditions (*11*).

Because FBT manifests with undifferentiated influenza-like symptoms that resemble other illnesses, cases are often overlooked or misdiagnosed by healthcare providers (*13*). Delayed or incorrect diagnosis might postpone necessary treatment, which can increase the risk for complications, prolonged illness, or, in severe cases, death (5). Such diagnostic challenges can also affect public health disease surveillance and understanding of disease effects.

Serologic testing for *R. typhi* by using indirect immunofluorescence antibody (IFA) assay is typical for diagnosis (*5*). Serologic testing performed on specimens collected within the first week of symptom onset can be falsely negative because detectable antibodies might not yet be present (*14*). Serologic confirmation of *R. typhi* infection requires paired testing of acute and convalescent specimens (drawn within 2–10 weeks) demonstrating a 4-fold increase in IgG titers. However, most patients do not return for second specimen collection. Real-time PCR testing can provide diagnostic confirmation earlier in the disease, when serologic testing might yield nonreactive or low-titer positive results. PCR is most sensitive when conducted on specimens collected within the first week of symptom onset and before the initiation of doxycycline, especially on whole blood or tissue specimens (*15*). Unfortunately, commercial PCR tests are not widely available for provider use to aid in the clinical diagnosis of FBT (*16*). Newer technologies for FBT diagnosis, such as Karius microbial cell-free DNA testing, provide additional alternatives for early detection, but their use is limited by accessibility and expense (*17*).

In California, FBT is a mandated reportable disease under Title 17, California Code of Regulations, §2500. LAC Department of Public Health (LACDPH) receives all positive *R. typhi* test results for LAC residents through electronic laboratory reporting or direct provider reports. LACDPH uses California Department of Public Health (CDPH) surveillance case definitions to determine if the report should be included in surveillance data to monitor disease burden and trends in LAC (Appendix Table).

Because FBT continues to increase locally, LACDPH implemented an enhanced protocol for additional testing of commercial laboratory low-titer positive *R. typhi* specimens from clinically compatible case reports. Those reports with low-titer positive serologic testing are not routinely investigated because they fall below the minimum laboratory criteria required for probable or confirmed CDPH case classification and are instead classified as suspected cases for internal tracking only. The goal of the enhanced protocol we describe in this article was to assess the extent to which routine investigative procedures missed true FBT cases among suspected cases and to determine the utility of molecular testing in supporting FBT clinical diagnosis and public health surveillance.

## Methods

In April 2022, LACDPH initiated an expanded testing protocol of patient specimens with low-titer positive *R. typhi* serology results reported from commercial laboratories. Inclusion criteria for this protocol were patients with low titer results of *R. typhi* IgG (1:64) or IgM (1:64 or 1:128) and symptoms consistent with the CDPH clinical criteria for FBT (Appendix Table). Patients who meet those criteria are considered suspected cases according to the CDPH surveillance definition because they have clinically compatible illness but insufficient laboratory evidence with serum titers below the required threshold values (IgG >1:128, IgM >1:256). We excluded low-titer positive specimens from this analysis if patients had a secondary specimen reported to LACDPH that met laboratory criteria for a probable or confirmed case. We requested and reviewed medical records for all patients to ensure that clinical criteria were met. Our study included patients with symptom onset dates during April 2022–December 2024.

LACDPH obtained serum specimens from patients meeting the study inclusion criteria and forwarded them to its public health laboratory (LACPHL) for repeat *Rickettsia* sp. IgG and IgM IFA serologic testing and to the CDPH Viral and Rickettsial Disease Laboratory for *Rickettsia* sp. nucleic acid detection by real-time PCR. During the study period, 3 real-time PCRs were used to test serum specimens because of changes in laboratory testing availability and procedures. We tested 13 specimens received through September 2022 by using a DNA-based pan-*Rickettsia* assay (*18*). During October 2022–April 2023, we tested 30 specimens by using a DNA-based (duplex) assay that targeted a 119-bp repeat region within the gene for surface cell antigen 2 (an autotransporter protein) (*11*). During May 2023–December 2024, we tested 70 specimens by using a triplex real-time reverse transcription PCR targeting regions of the 23S rRNA specific to *R. typhi*, *R. rickettsii*, and the *Rickettsia* genus (*19*). There was 1 sample we could not test. We entered and managed patient clinical and laboratory data by using REDCap (https://project-redcap.org) electronic data capture tools hosted at LACDPH (*20*,*21*). We collected data through medical records abstraction and patient interviews, and we included demographics, hospitalization, pertinent symptoms, blood chemistry results, FBT diagnostic testing, and doxycycline treatment. We conducted descriptive analyses of the study population and testing results by using SAS version 9.4 (SAS Institute Inc., https://www.sas.com). We also compared the study population with cases with high-titer positive specimens that met the CDPH criteria of probable or confirmed cases to assess for any major differences. We conducted significance analysis by using Fisher exact test for group comparisons of categorical variables and Wilcoxon rank sum test for group comparisons of continuous variables; we considered p<0.05 significant. Of note, we retrospectively tested all specimens originally tested by the pan-*Rickettsia* DNA assay and the duplex DNA assay with the newer rRNA assay by the CDPH Viral and Rickettsial Disease Laboratory to compare with prior results.

## Results

### Study Population Characteristics

LACDPH received 157 reports of patients with FBT low-titer positive test results; patients had symptom onset during April 2022–December 2024. We included 114 low-titer positive serum specimens in our study and retested the specimens for laboratory evidence of *R. typhi* infection. We did not include 43 low-titer positive serum specimens because those patients had high-titer positive convalescent serum specimens tested commercially (n = 23) or because specimens could not be obtained (n = 20). During the same period, high-titer positive results from 289 patients were reported to LACDPH (Table 1). We did not find significant demographic differences between low-titer and high-titer positive patients. For patients with low-titer positive results, the median age at diagnosis was 39 years (range 1–81 years). Fifty-three percent of the patients were male (n = 60) and 47% were female (n = 54). Most patients were ethnically Hispanic (61%, n = 69). Most patients were hospitalized (85%, n = 97) with a median stay of 5 days (range 1–23 days). After fever (required criteria), nausea or vomiting (69%, n = 79) and headache (67%, n = 76) were the most reported clinically compatible symptoms. Most patients (91%, n = 104), were treated with doxycycline; 66 patients (63%) were treated before or on the same day of specimen collection. More than half of the low-titer positive specimens in our study were collected 8–10 days after symptom onset (51%, n = 58); median time from symptom onset to date of specimen collection was 8 days (range 0–44 days). Low-titer positive patients were more likely to exhibit nausea or vomiting, rash, and thrombocytopenia than high-titer positive patients. Patients with low-titer positive results were more likely to be tested within the first week of illness onset than patients with high-titer positive results.

**Table 1:** Demographic and clinical characteristics of patients with possible fleaborne typhus, comparing 114 patients with initial low-titer positive serologic results with 289 patients with initial high-titer positive serologic results, Los Angeles County, California, USA, 2022–2024

Characteristics	Low-titer positive	High-titer positive
Age categories, y		
0–17	18 (16)	37 (13)
18–34	31 (27)	56 (19)
35–44	19 (17)	53 (18)
45–54	18 (16)	53 (18)
>55	28 (25)	90 (31)
Median age, y (range)	39 (1–81)	44 (2–87)
Sex		
M	60 (53)	177 (61)
F	54 (47)	110 (38)
Other/unknown	0 (0)	2 (0.7)
Race/ethnicity		
Hispanic/Latino	69 (61)	178 (62)
White	24 (21)	59 (21)
Asian	6 (5)	22 (8)
Other/unknown	15 (13)	22 (8)
Hospitalized	97 (85)	253 (88)
No. nights hospitalized, median (range)	5 (1–23)	5 (1–83)
Clinical characteristics		
Fever	114 (100)	289 (100)
Headache	76 (67)	195 (68)
Muscle pain/myalgia	69 (61)	155 (54)
Nausea/vomiting†	79 (69)	158 (55)
Rash†	57 (50)	93 (32)
Hematology results		
Thrombocytopenia, <150,000 platelets/µL†	87 (76)	177 (61)
Elevated ALT or AST, >40 IU/L	105 (96)	280 (98)
Treatment	104 (91)	244 (84)
No. days from specimen collection date to doxycycline treatment initiation, median (range)	0 (–3 to 31)	0 (–15 to 28)
Treated before or on same day of collection date	66 (63)	163 (67)
Treated >1 days after collection date	38 (37)	81 (33)
No. days from symptom onset date to specimen collection date†		
<7	41 (36)	50 (17)
8–10	58 (51)	101 (35)
>10	15 (13)	138 (48)
Median (range)†	8 (0–44)	10 (2–65)

*Values are no. (%) except as indicated. †p value <0.05 for this variable indicating significance.

### Additional Testing Results

Of the 114 retested specimens, repeat serologic testing on the same specimen at LACPHL resulted in a higher IgM or IgG titer in 106 (93%) of the specimens. Furthermore, 95 (83%) specimens resulted in IgG or IgM titers high enough to meet CDPH laboratory surveillance criteria for a probable case. Of the 113 specimens tested by PCR, 81 (71%) were PCR-positive, 22 (19%) were PCR-negative, and 10 (9%) had a PCR result of unsatisfactory or indeterminate. Specimens are considered inadequate for testing and reported as unsatisfactory if they did not meet required collection, shipping, or storage conditions upon arrival. An indeterminate result is reported when replicate results do not agree, which can occur when the gene target is near the limit of detection for the assay. All PCR-positive results were positive for *R. typhi*; no other *Rickettsia* species were detected. Of the 114 specimens, 113 (99%) came back with serologic testing or PCR results that changed their case classification from suspected to probable (28%, n = 32) or confirmed (71%, n = 81) (Table 2).

**Table 2:** Results of additional testing for initial 114 *Rickettsia* *typhi* low-titer positive serum specimens, Los Angeles County, California, USA, 2022–2024*

Category	No. (%) specimens
Initial commercial laboratory IFA *R.typh*i IgG titer	
<1:64	58 (51)
1:64	56 (49)
Initial commercial laboratory IFA *R. typhi* IgM titer	
<1:64	21 (18)
1:64	65 (57)
1:128	28 (25)
Repeat *R. typhi* IFA IgG titer	
<1:64	14 (12)
1:64	20 (18)
1:128	35 (31)
1:256	18 (16)
>1:512	25 (22)
Not done or unspecified	2 (2)
Total with IgG that met laboratory criteria (>1:128)	78 (68)
Repeat *R. typhi* IFA IgM titer	
<1:64	18 (16)
1:64	6 (5)
1:128	24 (21)
1:256	24 (21)
>1:512	38 (33)
Not done or unspecified	4 (4)
Total with IgM that met laboratory criteria (>1:256)	62 (54)
IFA serologic testing results higher upon repeat testing	106 (93)
IFA serologic testing results met laboratory criteria† upon repeat testing	95 (83)
PCR results	
Positive	81 (71)
Negative	22 (19)
Unsatisfactory or indeterminate	10 (9)
Not tested	1 (0.9)
Case classification change	
CDPH suspected to probable	32 (28)
CDPH suspected to confirmed	81 (71)
No change	1 (0.9)

*CDPH, California Department of Public Health; IFA, immunofluorescence assay. †IgG titer >1:128 or IgM titer >1:256.

### PCR Results by Timing of Specimen Collection and Serologic Findings

Among the 81 PCR-positive specimens, 34 (42%) were collected within 7 days of illness onset, 40 (49%) were collected at 8–10 days, and 7 (9%) were collected >10 days after onset. In contrast, among the 22 PCR-negative specimens, 4 (18%) were collected within 7 days of onset, 11 (50%) at 8–10 days, and 7 (32%) >10 days after onset. PCR-positive cases had a median of 8 days (range 1–15 days) and PCR-negative cases a median of 9 days (range 0–44 days) from illness onset to specimen collection. PCR-positive specimens were more likely than PCR-negative specimens to have IgM detected and no IgG detected (59%, n = 48) in the initial laboratory draw, whereas the opposite was observed for PCR-negative specimens (IgM not detected and IgG detected, 55%, n = 12) (Table 3).

**Table 3:** PCR results by timing of specimen collection and serologic findings of 103 specimens from patients with suspected *Rickettsia typhi* infection, Los Angeles County, California, USA, 2022–2024*

Characteristics	PCR positive, n = 81	PCR negative, n = 22
No. days from symptom onset date to specimen collection date†		
<7	34 (42)	4 (18)
8–10	40 (49)	11 (50)
>10	7 (9)	7 (32)
Median (range)†	8 (1–15)	9 (0–44)
Time from specimen collection date to doxycycline treatment start		
Treated before or on same day of collection	49 (64)	11 (65)
Treated >1 days after collection date	28 (36)	6 (35)
Initial commercial laboratory *R. typhi* IgG titer†		
<1:64	48 (59)	7 (32)
1:64	33 (41)	15 (68)
Initial commercial laboratory *R. typhi* IgM titer†		
<1:64	6 (7)	12 (55)
1:64	51 (63)	8 (36)
1:128	24 (30)	2 (9)
Initial commercial laboratory *R. typhi* results†		
IgM detected at 1:64 or 1:128 titer, IgG not detected	48 (59)	7 (32)
IgM not detected, IgG detected at 1:64 titer	6 (7)	12 (55)
IgM and IgG detected	27 (33)	3 (14)

*Values are no. (%) except as indicated. †p value <0.05 for this variable indicating significance.

### Retrospective Testing Results

Forty-three samples were initially tested by using DNA assays. We retested those samples by using the rRNA assay; 40 (93%) samples had concordant results, and 3 (7%) changed from unsatisfactory or indeterminate (n = 2) or negative (n = 1) to *R. typhi* positive.

## Discussion

In our study, most (71%) of the initial low-titer positive serology specimens obtained from patients with illness consistent with FBT were PCR-positive for *R. typhi*. That finding highlights the effectiveness of using PCR testing for clinical diagnosis and case detection of FBT. Unfortunately, *Rickettsia* sp. PCR testing has not been widely implemented because of the resources and laboratory expertise required (*14*). In the United States, rickettsial PCR testing remains largely limited to specialized public health laboratories and surveillance activities rather than routine clinical use (*22*).

Of note, most (91%) PCR-positive specimens were collected within 10 days after illness onset, with some positive up to 15 days. Those results suggest that the previously recommended 1-week window for FBT PCR testing might be too restrictive (*15*). Furthermore, our results suggest that rickettsial nucleic acids might persist in the bloodstream for longer than previously recognized, thereby extending the diagnostic window for molecular testing. Expanding the testing window beyond the first week could improve case detection and provide a more accurate understanding of the timing of rickettsial nucleic acid persistence in patients.

All 81 PCR-positive specimens in this study tested positive for *R. typhi* and none for *R. felis*. That finding reinforces a previous conclusion that *R. typhi* is the primary etiologic agent of FBT in California (*6*). Historically, both *R. typhi* and *R. felis* have been considered potential causes of FBT (*3*), including in urban Los Angeles, where both pathogens have been demonstrated to circulate in rat populations and associated fleas (*23*). The absence of *R. felis* in our PCR-positive specimens challenges previous assumptions about the involvement of different rickettsial species in FBT infection (*24*). Recognizing *R. typhi* as the principal agent is critical for interpreting the changing epidemiology of FBT, including increases in case numbers, geographic expansion, and evolving risk factors. Our results demonstrated variability in serologic testing results between commercial and public health laboratories. About 93% of the low-titer positive specimens obtained from commercial laboratories for repeat serologic testing had higher titers at the LACPHL. That result is a known major weakness of serologic testing, and our results align with existing evidence that the reading of IFA slides is prone to subjectivity (*14*). In addition, the lack of standardized IFA reagents, protocols, and criteria for test interpretation might account for titer variability between laboratories (*25*). In practice, it is not feasible for public health laboratories to repeat serologic testing of all commercial low-titer positive specimens. Obtaining confirmatory convalescent serology specimens is also not practical because many patients will have recovered by the recommended 10–14-day return window and no longer need medical care (*14*) or might face financial barriers preventing them from seeking further care. *Rickettsia* sp. serologic testing is also subject to high cross-reactivity between *Rickettsia* species (*16*). Because FBT manifests as a nonspecific illness, making PCR testing commercially available would enable rapid and accurate diagnosis of FBT and confirmation of *Rickettsia* species.

Nearly all (99%) specimens that were submitted for additional testing returned with results that met CDPH surveillance criteria for a probable or confirmed FBT case. Because of this testing protocol result, LACDPH has added 113 probable or confirmed cases of FBT from April 2022–December 2024 to overall case counts. Suspected cases are typically excluded from surveillance case counts publicly reported by public health jurisdictions. Public health jurisdictions that use a minimum titer to initiate case investigations might overlook those low-titer positive cases, resulting in underreporting of FBT case counts.

Our study demonstrates the importance of keeping IgM test results as part of the laboratory criteria in the FBT surveillance case definition. Studies suggest that *Rickettsia* IgM titers are less reliable and have limited utility compared with IgG titers because they have lower specificity and do not rise much earlier than IgG titers (*14*). Those studies prompted the exclusion of IgM test results from the CDPH and national FBT case definition laboratory criteria in 2025 (*26*). In our study, 48 (87%) of 55 patient specimens that had only IgM and no IgG detected in the initial testing by a commercial laboratory were found to be PCR-positive through public health testing. Those specimens were likely collected earlier in the disease course before a measurable rise in IgG titers occurred and when PCR testing is most sensitive. Those cases (n = 48) would have been misclassified if IgM testing results were not included in our protocol.

Most (91%) cases in this study were appropriately treated with doxycycline, demonstrating that clinicians are correctly suspecting FBT at the time of treatment initiation and ordering diagnostic testing. Furthermore, 63% of patients received doxycycline before or on the same day of specimen collection, indicating that patients with FBT in the differential diagnosis are being treated empirically before serology results are reported. That treatment pattern is encouraging because of the variability of rickettsial serology testing and aligns with public health messaging on the importance of early doxycycline initiation.

Our study population is representative of our overall FBT cases. The case demographics did not differ from those of high-titer positive cases. However, low-titer positive cases manifested with nausea or vomiting, rash, and thrombocytopenia more often than high-titer positive cases did. Low-titer positive cases were also more likely to be seen earlier in their illness, which might explain the differences in symptoms observed. The presence of those symptoms could lead persons to seek care earlier in the course of illness or clinicians to more readily consider FBT in the differential diagnosis.

Retrospective testing of specimens by using rRNA did not change our conclusion that most low-titer specimens were PCR-positive. The rRNA assay did detect 3 additional PCR-positive specimens that had previously returned unsatisfactory, indeterminate, or negative results by the DNA-based assays. Those results would have changed the classification of 3 cases from probable to confirmed. Those cases could not be reclassified in surveillance data because case investigations are finalized after the completion of each calendar year.

The first limitation of our study was the use of residual serum specimens for PCR testing, rather than whole blood or plasma, which are better suited for molecular detection of *Rickettsia*. Because *Rickettsia* are obligate intracellular organisms, their nucleic acids are more concentrated in cellular components and plasma compared with serum, where clot formation removes most cells and might reduce detectable DNA (*14*,*27*). Although only a small percentage of our specimens tested negative for *Rickettsia* nucleic acids by PCR, some of those results might represent false negatives attributable to the use of serum specimens. Of note, 1 case had both serum and plasma from the same collection date available for testing; in that case, *R. typhi* nucleic acids were detected in the plasma but not the serum, underscoring the effect of specimen type on PCR sensitivity. Second, the true effect of FBT in LAC is likely underestimated, and our findings might not fully represent the extent of disease transmission in the community. The reporting of FBT test results to LACDPH by laboratories and healthcare providers could be incomplete, resulting in possible missed cases. Patients with mild FBT symptoms who might not have sought care, were not tested, or were tested very early in their disease course, when antibody titers were undetectable, were not captured in this analysis. Because the study was geographically restricted to LAC, the findings might not be representative of the general population of the United States. Third, although LACDPH attempted to obtain all medical records relevant to the patients’ illness with FBT, it is possible that some follow-up visits were not captured, resulting in incomplete information on doxycycline treatment. Nonetheless, a high percentage of patients received doxycycline treatment in our study. Fourth, the sample size for subgroup comparisons was relatively small. Some statistical analyses, particularly those comparing PCR-positive and PCR-negative patients, had smaller subgroup sizes, reducing statistical power and increasing the likelihood of random error. Last, not all specimens could be obtained for analysis. Commercial laboratories generally retain specimens for a limited time, and retention times vary across laboratories. Delays in obtaining medical records to confirm that patients met clinical criteria meant that some specimens were discarded before they could be retrieved for study purposes, ultimately reducing the total number of specimens available for this analysis.

In conclusion, we demonstrate that many low-titer seropositive results from patients with clinically compatible illness represent true *R. typhi* infections and highlight the utility of molecular testing in supporting both clinical diagnosis and public health surveillance of FBT. PCR testing identified infections beyond the traditionally recognized 1-week window of PCR positivity and up to 15 days after symptom onset, demonstrating that molecular techniques can help confirm infection even into the second week of illness. Enhanced public health laboratory testing, like that conducted in our study, is effective for accurately classifying cases for comprehensive case surveillance. However, that approach is not sustainable as routine practice and further strains limited public health resources. Wider commercial availability of *Rickettsia* PCR testing and other new technologies could substantially enhance both clinical diagnostic capacity and our understanding of true disease effects. The resurgence of FBT in Los Angeles County and other areas of the United States highlights the importance of reexamining the current diagnostic protocols being used in clinical settings and public health surveillance.

Appendix Additional information about improved case detection of fleaborne typhus by PCR testing, Los Angeles County, California, USA, 2022–2024.
