# Supplementary material for: Improved Case Detection of Fleaborne Typhus by PCR Testing, Los Angeles County, California, USA, 2022–2024
Source: Emerg Infect Dis. 2026 Sep;32(9):1413–20. doi: 10.3201/eid3209.260554 (PMC13555722; doi:10.3201/eid3209.260554)
Supplement: Supplementary file 1 [file 26-0554-Techapp-s1.pdf]

Article DOI: https://doi.org/10.3201/eid3209.260554

EID cannot ensure accessibility for supplementary materials supplied by authors. Readers who have difficulty accessing supplementary content should contact the authors for assistance.

Improved Case Detection of Fleaborne Typhus by PCR Testing, Los Angeles County, California, USA, 2022–2024

Appendix

Appendix Table. Flea-borne typhus case definition developed by California Department of Public Health (CDPH) (2020)

Clinical Criteria (For the purpose of surveillance)	
Fever as reported by the patient or healthcare provider, AND two or more of the following: myalgia, headache, nausea/vomiting, elevated liver enzymes, rash, or thrombocytopenia.	
Laboratory Criteria for Diagnosis	
Confirmatory laboratory evidence	
• Detection of *R. typhi* nucleic acid in a clinical specimen via amplification of *R. typhi* target by rt-PCR assay, OR	
• Serologic evidence of a 4-fold increase in IgG-specific antibody titer reactive with *R. typhi* by IFA between paired serum specimens (one taken in the first 2 weeks of illness and a second up to 10 weeks later) and with the second serum sample having a titer of ≥1:128, OR	
• Demonstration of typhus fever group antigen in a biopsy or autopsy specimen by immunohistochemistry (IHC), OR	
• Isolation of *R. typhi* organisms from a clinical specimen in cell culture and molecular confirmation (e.g., PCR or sequence).	
Presumptive laboratory evidence	
• Has serologic evidence of elevated IgG at a titer of ≥1:128 reactive with *R. typhi* antigen by IFA in a sample taken within 60 d of illness onset, OR	
• Has serologic evidence of elevated IgM at a titer of ≥1:256 reactive with *R. typhi* antigen by IFA in a sample taken within 60 d of illness onset	
Epidemiologic Linkage Criteria	
A clinically compatible case that:	
• Was in the same household/same defined exposure as a confirmed case within the past 14 d before the onset of symptoms, OR	
• Likely had vector exposure in an area with suitable seasonal and ecologic conditions for potential vector-borne transmission	
Case Classification	
Confirmed:	A clinically compatible case (meets clinical criteria) that is laboratory confirmed.
Probable:	A clinically compatible case (meets clinical criteria) that has presumptive laboratory evidence and evidence of epidemiologic link.
Suspected:	A case with presumptive or confirmatory laboratory evidence of infection but no clinical information available, OR<br>A clinically compatible case (meets clinical criteria) that has evidence of epidemiologic link but no laboratory testing or equivocal results.
